# Ultrasound‐controlled drug release and drug activation for cancer therapy

**DOI:** 10.1002/EXP.20210023

**Published:** 2021-12-28

**Authors:** Li Tu, Zhihuan Liao, Zheng Luo, Yun‐Long Wu, Andreas Herrmann, Shuaidong Huo

**Affiliations:** ^1^ Fujian Provincial Key Laboratory of Innovative Drug Target Research School of Pharmaceutical Sciences Xiamen University Xiamen P. R. China; ^2^ DWI – Leibniz Institute for Interactive Materials Aachen Germany; ^3^ Institute of Technical and Macromolecular Chemistry RWTH Aachen University Aachen Germany

**Keywords:** cancer therapy, drug activation, drug release, mechanical force, ultrasound

## Abstract

Traditional chemotherapy suffers from severe toxicity and side effects that limit its maximum application in cancer therapy. To overcome this challenge, an ideal treatment strategy would be to selectively control the release or regulate the activity of drugs to minimize the undesirable toxicity. Recently, ultrasound (US)‐responsive drug delivery systems (DDSs) have attracted significant attention due to the non‐invasiveness, high tissue penetration depth, and spatiotemporal controllability of US. Moreover, the US‐induced mechanical force has been proven to be a robust method to site‐selectively rearrange or cleave bonds in mechanochemistry. This review describes the US‐activated DDSs from the fundamental basics and aims to present a comprehensive summary of the current understanding of US‐responsive DDSs for controlled drug release and drug activation. First, we summarize the typical mechanisms for US‐responsive drug release and drug activation. Second, the main factors affecting the ultrasonic responsiveness of drug carriers are outlined. Furthermore, representative examples of US‐controlled drug release and drug activation are discussed, emphasizing their novelty and design principles. Finally, the challenges and an outlook on this promising therapeutic strategy are discussed.

## Graphical abstract

This review presents a comprehensive summary of the current understanding of ultrasound‐sensitive drug delivery systems for controlled drug release and drug activation. The typical mechanisms and the main factors affecting the ultrasonic responsiveness of drug carriers are summarized. Representative examples of ultrasound‐controlled drug release and drug activation are highlighted.

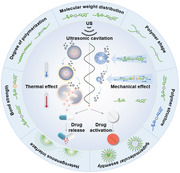

## 1 INTRODUCTION

Malignant tumors are perhaps the most frightening deadly diseases that threaten human health.^[^ ^1^ ^]^ Although clinical surgery has made impressive progress in alleviating pain and prolonging survival for cancer patients, the object of its services usually refers to early solid tumors.^[^ ^2^ ^]^ In the face of highly complex metastatic tumors, such physical resection seems impractical because of the infeasibility of its implementation in widespread metastatic lesions.^[^ ^3^ ^]^ Optimal treatment should be the combination use of chemotherapeutic drugs to inhibit the primary tumor and distant metastases.^[^ ^4^ ^]^ However, the high toxicity and non‐selectivity of conventional medicines commonly induce a trade‐off between desirable efficacy and harmful side effects.^[^ ^5^ ^]^ To overcome this contradiction, drug delivery strategies have been pursued by the scientific community for several decades, aiming to realize efficient and low‐toxicity cancer therapy.

Over the past few decades, rapid advances in nanotechnology have led to various active/passive targeted nano‐drug delivery systems (DDSs) being applied to increase drug accumulation in tumors while decreasing the cytotoxicity of drugs to normal cells.^[^ ^6^ ^]^ Nevertheless, the inefficiency of enhanced permeability and retention effects and the randomness of ligand‐receptor interactions make it difficult to achieve tumor‐specific targeted enrichment.^[^ ^7^ ^]^ Nor does Fick's law, which controls the diffusion of drugs, works only to designated cells, tissues, or organs.^[^ ^8^ ^]^ Moreover, there is growing recognition in clinical and preclinical research that cancer patients need not zero‐grade, sustainable drug release, but an on‐demand supply of therapeutic agents.^[^ ^9^ ^]^ Therefore, dosage‐, temporal‐, and spatial‐controlled DDSs have been exploited vigorously in recent years, which not only significantly heightens the selectivity of drugs but also effectively reduces their side effects.^[^ ^10^ ^]^

**FIGURE 1:**
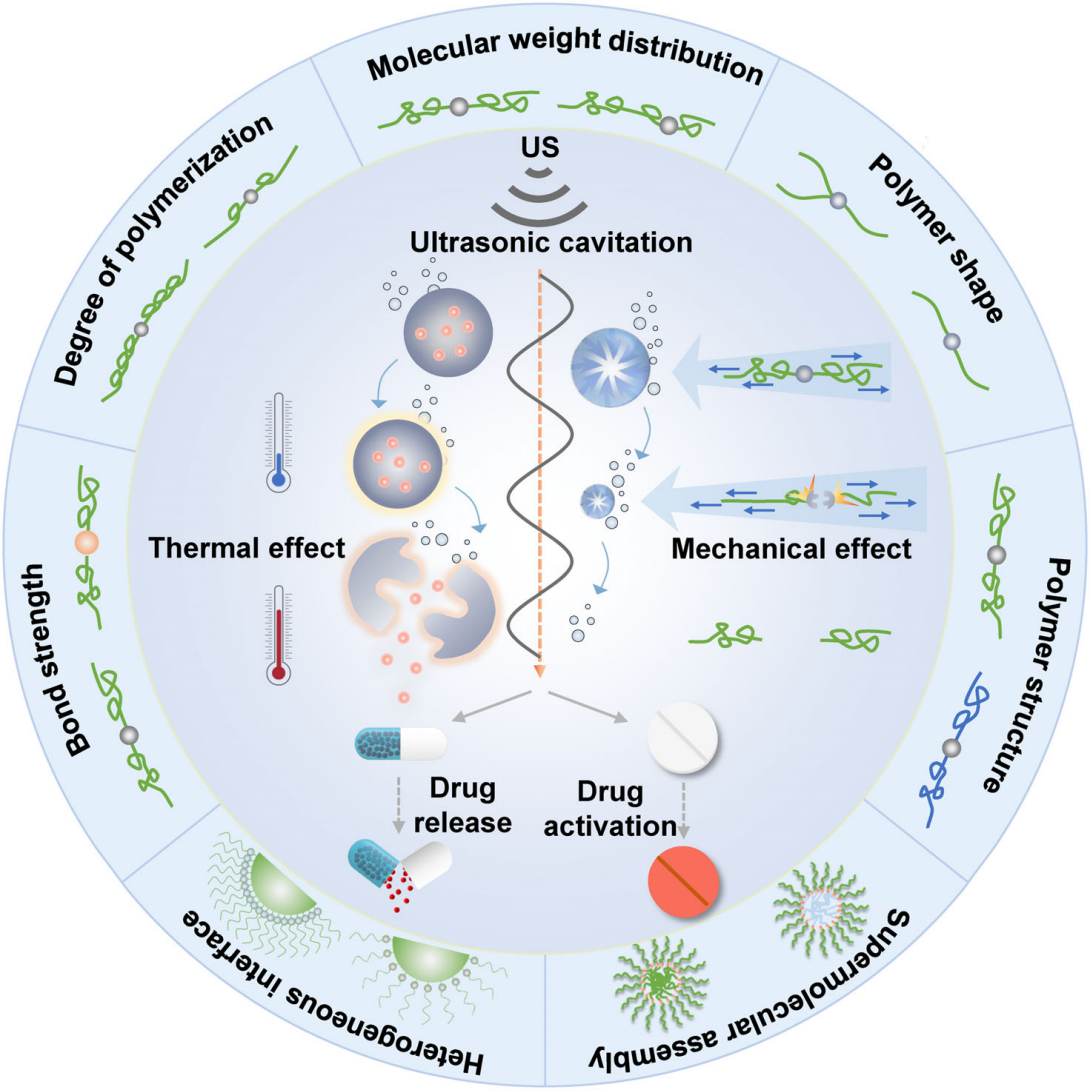
Schematic diagram of US‐controlled drug release and drug activation. US‐induced thermal and mechanical effects to control drug release and drug activation (inner ring). The structure–activity relationship of polymers on ultrasonic responsiveness (outer ring)

In general, the responsive DDSs are either sensitive to characteristic endogenous stimuli, such as pH, redox, enzymes, or ATP, or respond to external physical stimuli, including temperature, electromagnetic fields, light, or ultrasound (US).^[^ ^11^, ^12^ ^]^ Among them, US has many fascinating features, including non‐invasiveness, non‐ionizing radiation, high tissue penetration depth, and spatiotemporal controllability, for biomedical applications.^[^ ^13^, ^14^ ^]^ Particularly, US is considered an excellent tool for remote controlling drug release and activation, thereby preventing pharmacological toxicity from unnecessary damage to healthy tissues (Figure 1).^[^ ^15^ ^]^ Conceptually speaking, US is a mechanical wave of periodic vibration with frequencies beyond the range of human hearing (>20 kHz).^[^ ^16^ ^]^ In medicine, US with frequencies between 20 and 100 kHz is defined as low‐frequency ultrasound (LFUS), which is usually used to initiate sonophoresis and influence leakage of drug carriers through mechanical destruction.^[^ ^17^, ^18^ ^]^ Whereas high‐frequency ultrasound (HFUS) with frequencies greater than 1 MHz can reach deep into the tumor region by penetrating the skin and most tissues with a focused beam.^[^ ^19^, ^20^ ^]^ Meanwhile, HFUS can also prompt cellular uptake of therapeutics by temporarily increasing cell membrane permeability and further induce localized mild heat to trigger drug release from the vehicle.^[^ ^21^, ^22^ ^]^ More notably and most recently, polymer mechanochemistry utilizes the mechanical forces produced from US to regulate drug activity by breaking or rearranging labile bonds at the intended sites, offering a broad prospect for precise drug activation.^[^ ^23^ ^]^

This review summarizes the recent progress in selective control of drug release and drug activation by US for cancer therapy. First, we outline the typical mechanisms by using US for drug release and drug activation, including mechanical and thermal effects. Second, the main factors affecting the ultrasonic responsiveness of drug carriers are summarized, such as bond strength, molecular weight and degree of polymerization, molecular weight distribution, polymer shape and structure, supermolecular assembly, as well as heterogeneous interface. Furthermore, representative examples of US‐controlled drug release and drug activation are discussed, with an emphasis on their novelty and design principles. Finally, the challenges and perspectives in developing of US‐controlled DDSs for cancer treatment are provided.

## 2 MECHANISM OF US‐CONTROLLED DRUG RELEASE AND DRUG ACTIVATION

US‐responsive DDSs require biocompatible materials as carriers capable of releasing or activating therapeutic drugs through specific protonation, hydrolysis, phase transition, and molecular or supramolecular conformational changes. During this process, the pressure oscillation generated by US will affect the steady‐state of the drug carrier, and the accompanying mechanical and thermal effects are often more remarkable for the further conformational changes.

### 2.1 Mechanical effect

US‐induced mechanical effect is derived from stable cavitation generated by the continuous oscillation of microbubbles or inertial cavitation formed by rapid growth and bursting of microbubbles.^[^ ^24^ ^]^ Stable cavitation is generally caused by low amplitude (intensity or power) US. The continuous oscillation of the microbubbles creates velocities in the fluid that induce shear stress to destroy the carrier to release the encapsulated drug.^[^ ^25^ ^]^ At the same time, it also transiently forms holes through the cell membrane, resulting in the released drugs flowing into the cell.^[^ ^26^ ^]^ Inertial cavitation occurs when the intensity of US applied is high enough. Specifically, the collapse of microbubbles produces shock waves with amplitudes above 10,000 atmospheres.^[^ ^27^ ^]^ Despite the duration of the blast wave being short, the resulting pressure gradient is sufficient to damage the drug carrier with low mechanical strength, allowing it to release its cargos.^[^ ^28^ ^]^ In addition, collapsing microbubbles near the interface experience inhomogeneity, leading to the formation of high‐speed microjets.^[^ ^29^ ^]^ The shock waves resulting from microjets can enhance the permeability of cell membranes and blood vessels.^[^ ^30^, ^31^ ^]^ Noteworthy, the cavitation on the mechanical stimulation of blood vessels is also conducive to drugs across the blood‐brain barrier (BBB) for treating central nervous system related diseases.^[^ ^32^ ^]^

Instead of releasing the physically trapped drug through mechanical destruction, US can activate drugs by breaking the mechanochemical unstable bond within the carriers. In polymer mechanochemistry, collapsing microbubbles generated by acoustic cavitation create a mechanical elongational flow, stretching polymer chains and eventually inducing the breakage.^[^ ^33^ ^]^ Thus, the chemical properties of some polymers can be modulated at the molecular level.^[^ ^34^ ^]^ Primordially, the concept was originated from the discovery of mechanical degradation of polymers by Staudinger et al. in the 1930s.^[^ ^35^, ^36^, ^37^, ^38^ ^]^ Later in 1989, Langer et al. found that the US could prompt the release of small molecules in long chains of macromolecules.^[^ ^39^ ^]^ Simply speaking, a mechanic‐sensitive polymer needs the introduction of a mechanophore, a force‐sensitive molecular unit with a mechanically unstable bond, a tension ring, or an isomerization bond, into the polymer chain.^[^ ^40^ ^]^ As Moore et al. reported in 2005, functionalized polyethylene glycol (PEG) was selectively split at the weak azo bond in the chain center in response to the US.^[^ ^41^ ^]^ Similarly, peroxide (O─O) bond, coordinate bond, and disulfide (S─S) bond with low bond dissociation energy are also prone to cleavage when subjected to consecutive molecular strain.^[^ ^42^, ^43^, ^44^ ^]^ In contrast, strong bonds with high dissociation energy, such as carbon–carbon (C─C) bond, carbon–oxygen (C─O) bond, and carbon–nitrogen (C─N) bond, are challenging to be cleaved by ultrasonic mechanical force (sonomechanical force).^[^ ^45^ ^]^

### 2.2 Thermal effect

During the ultrasonic wave propagation in the medium, part of the acoustic energy will be absorbed by the medium and converted into heat.^[^ ^46^ ^]^ Besides, acoustic cavitation can result in the other two forms of thermal effect. One is the continuous thermal effect arised from the sustained oscillation of cavitation bubbles, which can cause the thermal energy deposition in the sound field region.^[^ ^47^ ^]^ The other is the instantaneous thermal effect, that is, the sudden collapse of the cavitation bubble results in local overheating.^[^ ^48^ ^]^ The temperature elevation usually occurs when the parameters of focused ultrasound (FUS) or high intensity focused ultrasound (HIFU) are set at moderate sound pressures, prolonged irradiation times, and high duty cycles.

Considering the accidental damage to surrounding normal cells caused by long‐term hyperthermia, US‐responsive thermosensitive DDSs should maintain stable at physiological temperature (∼37 °C), while rapidly releasing the drugs within the tumor (∼40 to 42 °C) heated locally by US.^[^ ^49^ ^]^ It requires that at least one component of the carrier material could be changed quickly and non‐linearly with temperature increase. Such DDSs typically refer to liposomes, nanoparticles (NPs), or polymer micelles exhibiting low critical solution temperatures.^[^ ^50^, ^51^, ^52^ ^]^ In terms of liposomes, acoustic‐thermal sensitivity generally results in phase transitions of lipid composition or conformational changes of the lipid bilayer.^[^ ^53^, ^54^ ^]^ For example, Dreher et al. synthesized a low‐temperature‐sensitive liposome (LTSL) containing lysolecithin lipid for delivery of the chemotherapeutic drug doxorubicin (DOX).^[^ ^55^ ^]^ Upon irradiation by HIFU, the LTSL dissolved once the temperature exceeds its phase transition temperature (∼40 to 42 °C). The result showed that, after HIFU irradiation, the DOX concentration in tumors treated with LTSL was 3.4‐fold than that without HIFU.

## 3 FACTORS AFFECTING THE ULTRASONIC RESPONSIVENESS

US‐responsive DDSs should be as sensitive to the external US as possible to mitigate undesirable tissue bioeffects aroused by sound pressure, acoustic cavitation, and acoustic heating.^[^ ^56^, ^57^ ^]^ Thus, a detailed understanding of the fundamental factors that control the ultrasonic sensitivity of carrier materials is essential to broaden their biomedical applications. This section will discuss the structure–activity relationship of bond strength, molecular weight and degree of polymerization, molecular weight distribution, polymer shape and structure, supermolecular assembly, and heterogeneous interfaces with US responsiveness, respectively.

### 3.1 Bond strength

**FIGURE 2:**
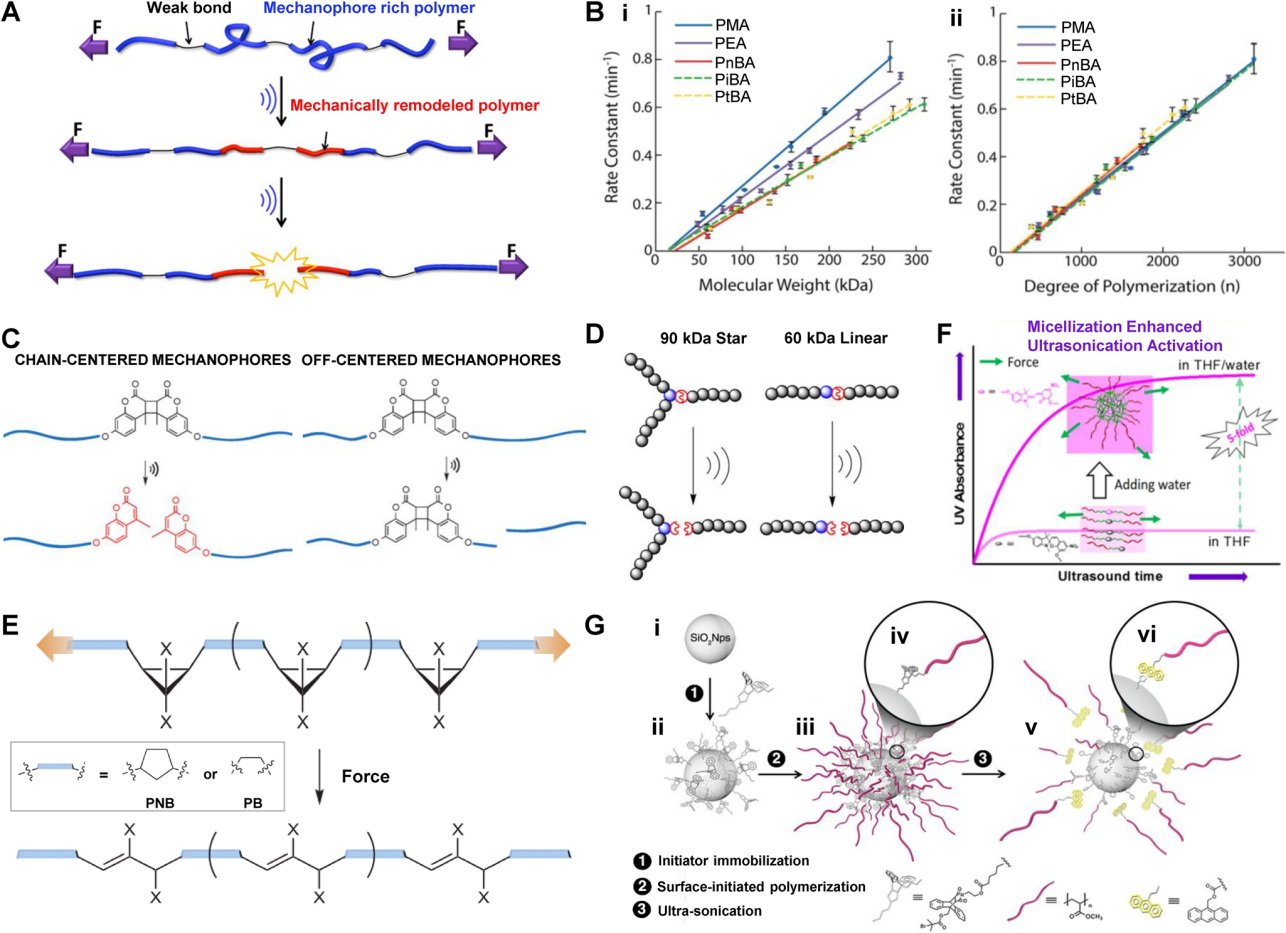
Factors affecting ultrasonic responsiveness of carrier materials. (A) Effect of bond strength on mechanochemical activation. Reproduced with permission.^[^ ^59^ ^]^ Copyright 2015, American Chemical Society. (B) (i) Relationship of molecular weight and (ii) degree of polymerization to mechanochemical activation of polymers. Reproduced with permission.^[^ ^62^ ^]^ Copyright 2016, American Chemical Society. (C) Influence of molecular weight distribution on scission of mechanophore. Reproduced with permission.^[^ ^65^ ^]^ Copyright 2015, Royal Society of Chemistry. (D) A general depiction of chain breaks in polymers of different shapes. Reproduced with permission.^[^ ^71^ ^]^ Copyright 2014, American Chemical Society. (E) Molecular structure affects polymer mechanochemistry. Reproduced with permission.^[^ ^74^ ^]^ Copyright 2013, Springer Nature. (F) Ultrasonic response of symmetric copolymer nanomicelles. Reproduced with permission.^[^ ^80^ ^]^ Copyright 2015, American Chemical Society. (G) Response of polymer‐grafted NPs to mechanical activation. Reproduced with permission.^[^ ^86^ ^]^ Copyright 2014, American Chemical Society

Mechanical bond fracture is the source of mechanoresponsive‐polymer failure, so it is reasonably necessary to explore the mechanochemical behavior of chemical bonds with various bond strengths.^[^ ^58^ ^]^ Representatively, employing the force response of non‐scissile gem‐dichlorocyclopropane (gDCC) mechanophores embedded in the polymer, the relative mechanical strengths of a series of scissile weak bonds were compared, including the C─N bond (24–30 kcal/mol) of azodialkylnitrile, the C─S bond (71–74 kcal/mol) of thioether, and the C─O bond (52–54 kcal/mol) of benzyl phenyl ether (Figure 2A).^[^ ^59^ ^]^ In this work, the degree of activation of non‐scissile gDCC mechanophores provides criteria for measuring the mechanical strength of these weak bonds under US. In simple terms, the fewer ring‐opening events that occur in gDCC mechanophores triggered by the pulsed US formed along the backbone, the more vulnerable the weak bonds are to break. Notably, a statistical analysis of the amount of ring opening of gDCC mechanophore displayed that the mechanical strengths of the three weak bonds are C─N bond (weakest) < C‐S bond < C─O bond (strongest), which are not consistent with their thermodynamic strength. The rehybridization caused by C─O bond fracture leads to the deterioration of mechanochemical coupling, which is the main reason for the increase of mechanical strength. Thus, the choice of mechanophore should not only consider the thermodynamic strength but also comprehensively analyze the consequences of secondary structure changes.

### 3.2 Molecular weight and degree of polymerization

The attachment of the polymer chain is a crucial part of transferring the mechanical force to the mechanophore.^[^ ^60^ ^]^ Early studies denoted that chain length was a key element determining the breakdown of polymer chains.^[^ ^61^ ^]^ In general, the chain length of polymers depends on their molecular weight and degree of polymerization. To choose a better descriptor of mechanochemical transduction in polymer, Moore et al. synthesized five kinds of acrylate monomers with different ester substituents that could be served as side‐chain units (Figure 2B).^[^ ^62^ ^]^ Since different compositions or structures of side chains could cause polymers with similar molecular weights but have varying degrees of polymerization, the mechanochemical effects of molecular weight and degree of polymerization might be appraised independently. Owning to its US‐induced mechanochemical ring‐opening transformation accompanied by absorption change, the spiropyran was chosen as an auxiliary for quantifying mechanochemical activity in this study.^[^ ^63^ ^]^ For each of the five discrepant polymer chains (i.e., PMA, PEA, PnBA, PiBA, and PtBA), the activation rates increased linearly with the augment of molecular weight upon US irradiation. Meanwhile, polymer chains with the same molecular weight exhibited quite different levels of activation. In comparison, polymer chains with the same degree of polymerization but diverse molecular weights exhibit similar ultrasonic transduction efficiency, suggesting that the degree of polymerization rather than molecular weight is the better descriptor of US‐induced mechanochemical transduction.

### 3.3 Molecular weight distribution

Ordinarily, ultrasonic degradation of homopolymer in solution is initiated from or near the center of the chain, where the stress tends to accumulate under elongational flow.^[^ ^64^ ^]^ Hence, the mechanophore is always arranged to reside in the middle of the chain to equalize the molecular weight. This in turn evoked curiosity about how the molecular weight distribution (i.e., polydispersity index, PDI) affects the mechanochemical activity. To this end, Craig et al. proposed a coumarin dimer (CD) mechanophore to produce a fluorescent coumarin chain‐end polymer to quantify mechanochemical activation efficiency.^[^ ^65^ ^]^ As expected, polymers with high PDI values have a significant likelihood of forming off‐centered mechanophores, which may easily lead to random breakage of molecular chains at non‐specific sites. A higher proportion of fractures occurred at CD when the relative molecular weight distribution was narrower, possibly because a lower PDI value was deemed to satisfy the condition that the mechanophore was located at the chain center (Figure 2C). Additionally, with the increase of the proportion of CD units at the chain center, the activation efficiency increased simultaneously, manifesting that the distance between the mechanophore and the chain center could be one of the independent variables that interfere with the activation efficiency. The results can also be explained as that the force enhances near the center of the polymer chain and attenuates as the distance from the center increases.^[^ ^66^, ^67^ ^]^ Therefore, molecular weight distribution is another subtle but important consideration in designing and fabricating mechanochemical responsive systems.

### 3.4 Polymer shape and structure

The transformation of polymer shape will bring about the variation of molecular weight distribution, which will affect the US‐induced chain‐breaking rate. For example, star‐shaped polymers show enhanced shear stability than linear polymers with the same molecular weight.^[^ ^68^, ^69^ ^]^ This result is attributed to star polymers’ low effective molecular weight, whose multi‐branched structure offsets part of the molecular weight, resulting in a low chain fracturing rate.^[^ ^70^ ^]^ Similarly, Boydston et al. synthesized a series of well‐defined linear and three‐arm polymers, and demonstrated that the fracture rate of the mechanophore is determined by the effective molecular weight rather than the total molecular weight (Figure 2D).^[^ ^71^ ^]^ Thus, it is not surprising that the activation rates of the three‐arm star polymer and their linear polymer analogs are consistent, even though the molecular weight of the star polymer is 1.5 times that of the linear polymer.

Theoretically, the sonomechanical effect on the mechanophore embedded in the polymer backbone hinges not only on the shape of the polymer chain but on its structure.^[^ ^72^, ^73^ ^]^ In a related study, single molecular force spectra were directly used to quantify the ring‐opening forces of gem‐dibromo and ‐dichlorocyclopropane fixed along with the skeletons of *cis*‐polynorbornene (PNB) and *cis*‐polybutadiene (PB) (Figure 2E).^[^ ^74^ ^]^ The critical force for isomerization of PNB scaffolding was decreased by about one‐third compared to the PB framework due to the more efficient mechanochemical coupling of the PNB backbone, which allows it to act as a lever to enhance polymer mechanochemistry.

### 3.5 Supermolecular assembly

US activation of mechanophores is often unsatisfactory because of the high threshold molecular weight for launching mechanochemical activation.^[^ ^75^, ^76^ ^]^ The conventional method of ameliorating the activation of the mechanophore is to increase the effective molecular weight of the polymer chain to improve the degree of polymerization at both ends of the mechanophore, which is impeded by the complexity of synthetic methods. It has been reported that the mechanophore in the entangled or swollen state has higher mechanochemical activity than in the stretched form.^[^ ^77^, ^78^, ^79^ ^]^ Enlightened by this, Du et al. assembled amphiphilic symmetric triblock polymers ([spiropyrane‐(t‐BA_88_‐*b*‐NIPAM_62_)_2_], P2) into micelles with entangled chain as the core and stretched chain as the outer shell through supramolecular self‐assembly technique (Figure 2F).^[^ ^80^ ^]^ Due to the fundamental variations in the physicochemical properties of the micellated polymer chains, the sonomechanical force transmitted along the polymer chain has more favorable mechanochemical transduction, which is entirely different from that of its dissolved counterpart. By characterizing the UV‐Vis absorption of merocyanines from the conversion of spiropyran, they observed that the mechanical response of P2 micelles was five times higher than that in the stretched state. Moreover, the mechanical responsiveness was improved dramatically with the exaltation of micellar degree. The reason could be the increasing micellization degree makes the spiropyran closer to the solidified state, which has better mechanical sensitivity than dissolution. Given this study, the mechanophore could display high mechanochemical activity even in relatively low molecular weight chains. In addition, recent studies have found that temperature and solvent are two major regulatory factors affecting ultrasonic responsiveness, as they determine the thermodynamic state (metastable or stable) of the nanoassembly.^[^ ^81^, ^82^ ^]^ In general, metastable polymer micelles assembled in a specific solvent have better ultrasonic responsiveness than their stable state. Also, as the self‐assembly temperature approaches the glass transition temperature, the ultrasonic responsiveness gradually increases due to the improved mobility of the polymer chain.

In another study, Heuts et al. synthesized block copolymers consisting of long hydrophilic polyacrylic acid (PAA) and short hydrophobic polybutyl acrylate (PBA) linked by an anthracene‐maleimide Diels‐Alder (DA) adduct.^[^ ^83^ ^]^ Unlike the symmetrical block polymers described above, the short‐chain of PBA makes it challenging to form serviceable tangles at the self‐assembled micelle core.^[^ ^84^ ^]^ Even so, the hydrophilic PAA chains still cleaved from the block copolymers after sonication. This could be due to the establishment of micellar nuclei with a much higher viscosity than water under the non‐covalent interaction between PAA and PBA, thus enlarging the profile length of supramolecular aggregates and augmenting the acting area of the elongational force. The availability of relatively short PBA chains indicated that these DA adducts do not need to be entangled before activation. Indeed, since micellar aggregation is the driving force for mechanochemical activation, partial mechanophores cannot be cleaved if the polymer is not assembled into a micelle structure. Consequently, supramolecular scaffolds such as micelles are practical tools for ameliorating the mechanochemical activity of the mechanophore.

### 3.6 Heterogeneous interface

So far, the exploration of mechanophores has focused on homogeneous systems in which the bond breakage occurs at the center of the polymer chain.^[^ ^85^ ^]^ Considering the growing application of composite materials in biomedical fields, the mechanochemical behaviors at the heterogeneous interface should not be neglected. Using cycloadduct of maleimide‐anthracene (MA) as model mechanophores, Moore et al. prepared MA‐anchored poly(methyl acrylate) (PMA) grafted silica NPs (SiO_2_ NPs‐MA‐PMA) to verify the selective activation of mechanophores at heterogeneous interfaces (Figure 2G).^[^ ^86^ ^]^ By synthesizing a series of PMA chains with different molecular weights, they found that the activation of the polymer‐bound mechanophores is still molecular weight dependent. Also, the threshold molecular weights at the heterogeneous interface that can activate SiO_2_ NPs‐MA‐PMA are similar to those of their homopolymer counterparts. By comparing the morphological variation of SiO_2_ NPs‐MA‐PMA before and after US, the interface between the polymer and NPs obviously isolated after the mechanophore was activated. Besides, the NPs were changed from hexagon to irregular shape, which is likely due to the PMA chain fracture that affected ester group anchored on SiO_2_ NPs. More importantly, the results provide theoretical basics for expanding the polymer mechanochemistry study to the nanoscale domain.

After ascertaining the activation potential of the mechanophore at heterogeneous interfaces, the researchers investigated the effect of graft density on the mechanochemical activity.^[^ ^87^ ^]^ Based on the MA‐mechanophore, SiO_2_ NP_S_‐MA‐PMA with grafting densities of 0.27, 0.18, and 0.05 chain/nm^2^ were synthesized. It should be noted that, for the same molecular weight, the system with the lowest graft density exhibited the most robust mechanochemical activity, while the system with the highest graft density showed the lowest activation rate. Moreover, when the graft density decreased by 37%, the mechanochemically activated retro cycloaddition was accelerated distinctly, which was equivalent to an additional increase of 10 kDa in molecular weight. In other words, the threshold molecular weight in connection with mechanical activation decreases as the graft density decreases, which is in sharp contrast to the studies mentioned above. The difference is that the polymer in this work is in a highly stretched state. Further increase in graft density contributes to enhanced interactions between adjacent chains, including interchain entanglement, which dilutes the concentration distribution of forces in the polymer chain, thereby preventing the polymer chain from breaking.^[^ ^88^ ^]^ Overall, graft density is like a double‐edged sword that needs to be considered comprehensive, and the stretched state of the polymer chain could eventually dominate the mechanochemical activity at the heterogeneous interface.

## 4 US‐CONTROLLED DRUG RELEASE

With the incremental understanding of the factors affecting the ultrasonic responsiveness of carrier materials, a wide variety of US‐responsive DDSs have been triumphantly constructed.^[^ ^89^ ^]^ Encapsulating drugs in these DDSs allows them to be immune to the surrounding physiological environment and only work on specific sites controlled by US. This section will pay attention to nano‐micro systems triggered by US for controlled drug release, including microbubbles, liposomes, and silicon‐based NPs.

### 4.1 Microbubbles

Microbubbles are inflatable spheres (1–8 μm) dispersed in an aqueous medium, which have been extensively used as contrast agents for US imaging.^[^ ^90^, ^91^ ^]^ More importantly, the collapse of microbubbles can be used to control drug release by reducing the cavitation threshold.^[^ ^92^ ^]^ When stimulated by a near‐resonant US frequency, the microbubble oscillates like a cavitation bubble, which may burst in the end.^[^ ^93^ ^]^ Similar to cavitation nuclei, microbubbles can strengthen energy deposition in tissues and cells, temporarily destroying the cell membrane integrity through sonoporation, thereby facilitating intracellular drug transportation.^[^ ^94^ ^]^

A representative example of microbubbles in drug delivery is to enhance the targeted treatment of brain tumors. The existence of the blood‐brain barrier (BBB) results in difficulties of drug spreading through the bloodstream to the central nervous system.^[^ ^95^ ^]^ Fortunately, microbubbles can open the BBB without damage by loosening the tight junctions of capillary endothelial cells under acoustic cavitation.^[^ ^96^ ^]^ For instance, Yeh et al. designed a microbubble (DOX‐SPIO‐MB) containing iron oxide NPs and DOX for imaging‐guided treatment of brain tumors.^[^ ^97^ ^]^ The superparamagnetic and acoustic properties of DOX‐SPIO‐MB enable the system to guide tumor therapy in real‐time through magnetic resonance (MR) and US imaging. To improve the delivery efficiency of DOX to cerebral blood vessels, a brain‐targeting ligand was modified on the surface of microbubbles.^[^ ^98^ ^]^ The results showed that the BBB was successfully opened simultaneously as the acoustic cavitation‐induced drug release, which expedited drug delivery and enhanced the therapeutic effect.

**FIGURE 3:**
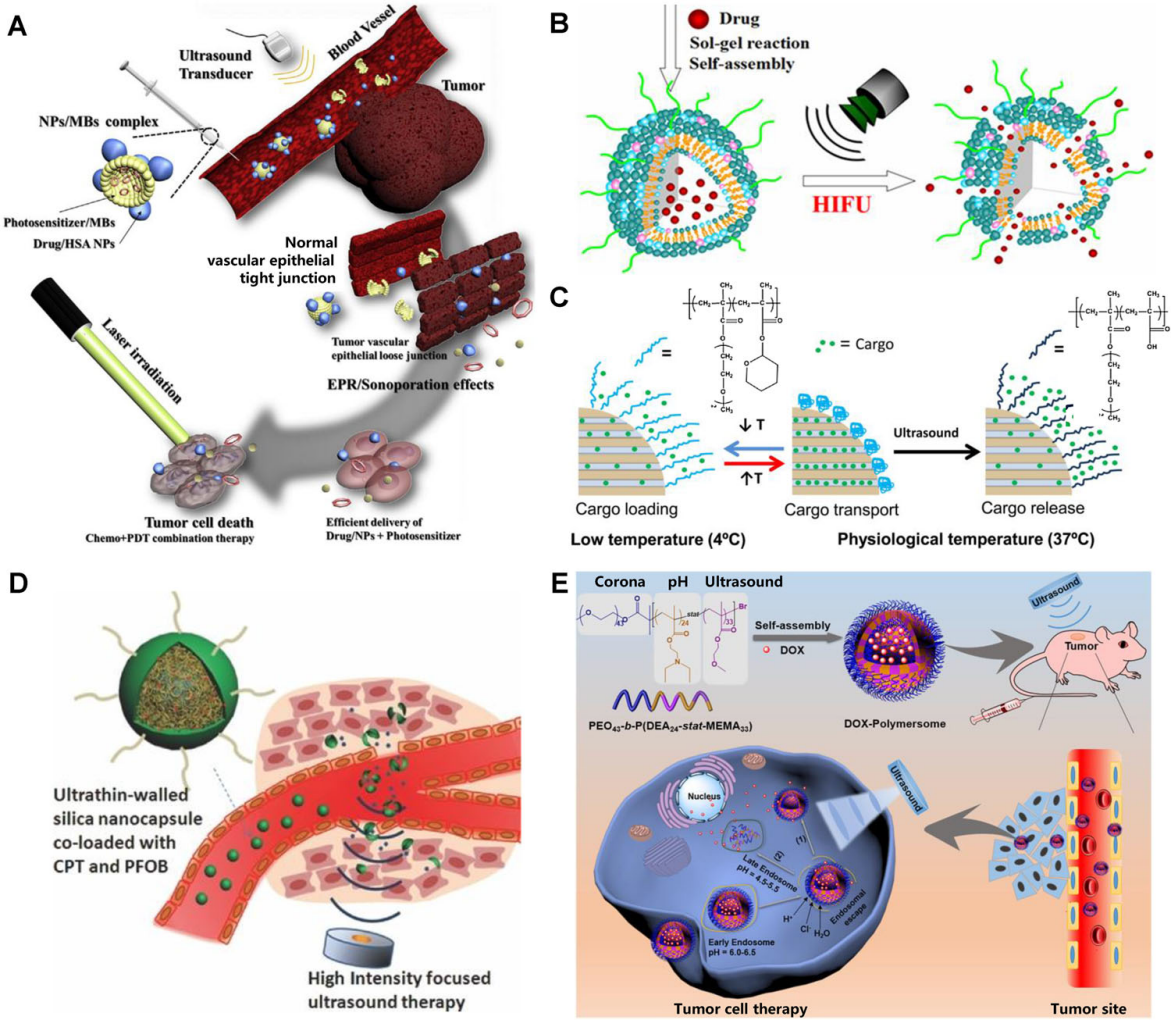
Representative examples of US‐controlled drug release. (A) US‐triggered drug release based on DOX‐NPs/Ce6‐MBs complex. Reproduced with permission.^[^ ^102^ ^]^ Copyright 2018, Elsevier. (B) Drug release from HTSC under HIFU sonication. Reproduced with permission.^[^ ^108^ ^]^ Copyright 2015, American Chemical Society. (C) Drug release from polymer‐grafted MSNs triggered by US heating. Reproduced with permission.^[^ ^113^ ^]^ Copyright 2015, American Chemical Society. (D) HIFU‐induced silica shell cracking of CPT/PFOB@SNCs for controlled release of CPT. Reproduced with permission.^[^ ^116^ ^]^ Copyright 2014, John Wiley & Sons. (E) US‐responsive polymersomes for facilely controlled drug delivery. Reproduced with permission.^[^ ^119^ ^]^ Copyright 2020, Elsevier

Apart from delivering chemotherapeutic agents, microbubble can also be used to load photosensitizers for synergistic chemotherapy and photodynamic therapy (PDT). It has been reported that reactive oxygen species (ROS) produced by PDT can motivate intracellular drug transport, accelerate drug release, and elevate cytotoxicity.^[^ ^99^, ^100^, ^101^ ^]^ Kim et al. fabricated a composite system (DOX‐NPs/Ce6‐MB) consisting of DOX wrapped in human serum albumin NPs and chlorin e6 (Ce6) encapsulated in microbubbles, in which the NPs were bound to the surface of microbubbles for US‐triggered local drug delivery (Figure 3A).^[^ ^102^ ^]^ Once US was applied, the microjet generated by the explosion of microbubbles can boost the penetration of the locally released NPs into the tumor. Under subsequent laser irradiation, Ce6 induces a large amount of ROS production in tumor cells for synergistically enhanced chemotherapy for anti‐tumor therapy.

### 4.2 Lipidosomes

The relatively large size of the microbubbles leads to a short lifespan, which makes them unsuitable for tumor targeting and retention.^[^ ^103^, ^104^ ^]^ Encouragingly, benefiting from the advantages of NPs, such as small size, large specific surface area, good pharmacokinetics, excellent targeting ability, and accessible surface functionalization, various nano‐DDSs with controllable drug release are highly favored.^[^ ^105^, ^106^, ^107^ ^]^ Among them, US‐responsive thermosensitive liposome (TSL) is one of the typical representatives. For example, Dai et al. transformed ceramic liposomes into HIFU‐responsive liposomes (HTSC) by using sol‐gel reactions and self‐assembly techniques (Figure 3B).^[^ ^108^ ^]^ Owing to the surface coating of organosiloxane atoms, HTSC has better structural stability than conventional TSL, preventing the drug's early leakage. Under the high temperature generated by HIFU irradiation, the cumulative release of DOX encapsulated in the HTSC could be reached to 90%, strikingly suppressing the tumor growth.

For non‐thermoresponsive liposomes, Yechezkel et al. demonstrated the feasibility of controlling drug release using LFUS‐induced mechanical effects.^[^ ^109^ ^]^ They first confirmed a positive correlation between liposomal drug release and ultrasonic amplitude. When the irradiation amplitude was in the range of 0–1.3 W/cm^2^, the cumulative drug release manifested a relatively slow linear increase. Once the irradiation amplitude was upregulated, the drug release rate was markedly improved about four times, which could be interpreted as the initiation of inertial cavitation. Noteworthy, the drug release curves for continuous or pulsed LFUS irradiation were roughly the same for the same irradiation time, implying that drug release only depended on the actual exposure time. Afterward, they further verified the effectiveness of cisplatin‐loaded liposomes for local drug delivery in tumors.^[^ ^110^ ^]^ The results revealed that nearly 70% of cisplatin was released in tumors exposed to LFUS, but only 3% of drug release was observed in the group without LFUS. Since multi‐interval US is helpful to avoid interrelated thermal damage, which is superior to single‐use continuous US, the study is beneficial for the clinical applications of US‐based drug delivery strategies.

### 4.3 Silicon‐based NPs

Compared with liposome‐based DDSs, mesoporous silica NPs (MSNs) has more robust structural stability under physiological conditions.^[^ ^111^ ^]^ Nevertheless, the weak affinity between hydrophobic drugs and the silanol group in the mesoporous channel may lead to low drug loading and premature drug leakage.^[^ ^112^ ^]^ Considering that 2‐tetrahydropyranic methacrylate (THPMA) is a hydrophobic monomer with an unstable aldehyde group, which can be cleaved to hydrophilic methacrylate (MAA) by acoustic heating. Vallet‐Regıí et al. coupled a THPMA‐containing copolymer p(MEO_2_MA‐*co*‐THPMA): poly(2‐(2‐methoxyethoxy) ethyl methacrylate‐*co*‐THPMA to the surface of MSNs, making it as a gatekeeper to control the drug release (Figure 3C).^[^ ^113^ ^]^ In the typical physiological environment, the copolymer exhibited a collapsed state on the surface of MSNs, and the drugs were utterly trapped in the micropores. Upon HFUS irradiation, the copolymer was pyrolyzed and cleaved into p(MEO_2_MA‐MAA) and tetrahydropyranol, which became more hydrophilic to open the gates and release the captured DOX.

Except for the gating strategy, Chen et al. prepared an organic‐inorganic hybrid framework to intensify the interactions between mesoporous organosilicone and drug molecules.^[^ ^114^ ^]^ Various organic molecules can be integrated into the skeleton of silica (SiO_2_) through non‐covalent interactions, including electrostatic and hydrophobic interactions. For example, MSN with phenyl‐conjugated hybridizations has strong adsorption of hydrophobic paclitaxel (PTX), leading to a high drug loading.^[^ ^115^ ^]^ After external US irradiation, the non‐covalent interaction between the drug and the carrier was easily interrupted meantime accelerating the drug release. It is worth noting that the drug release strategy is reversible, and controlled pulsatile drug release can be achieved by switching ON/OFF of HIFU.

Owning to its high mechanical strength and heat resistance property, SiO_2_ can be used as a skeleton to load drugs and as a covering layer to prevent premature drug leakage. For example, Shi et al. manufactured a multifunctional nano‐DDS for HIFU‐triggered drug release and synergistic tumor ablation (Figure 3D).^[^ ^116^ ^]^ The chemotherapeutic drug camptothecin (CPT) and US‐sensitive perflubron (PFOB) were encapsulated in poly(lactic‐*co*‐glycolic acid) (PLGA) nanocapsules. Afterward, ultrathin SiO_2_ was functionalized as a shell to prevent CPT from escaping before reaching the tumor. Once the nanosystem is subjected to HIFU, the encapsulated PFOB can be transformed into microbubbles by the vaporization of acoustic droplets. As a result, the produced microbubbles effectively reinforced the acoustic cavitation and directly smashed the SiO_2_ coating through mechanical effect, promoting the explosive release of CPT.

### 4.4 Polymer‐based NPs

Polymer‐based NPs have been widely used for remote control of drug release in cancer therapy due to their excellent physiological stability and ultrasonic responsiveness. Among them, polymer vesicles and micelles are two typical representatives.^[^ ^117^ ^]^ Earlier, Du et al. reported a polymer vesicle composed of poly(ethylene oxide)‐*block*‐poly[2‐(diethylamino)ethyl methacrylate‐stat‐2‐tetrahydrofuranyloxy)ethyl methacrylate].^[^ ^118^ ^]^ After US irradiation, the size of these vesicles was significantly reduced due to the rapid rupture and recombination of the vesicle membrane. The ^1^H NMR spectra showed that physical but not chemical changes took place during the reassembly process. In a follow‐up study, they further developed a novel US‐responsive vesicle for controlled drug release via self‐assembly of block copolymer poly(ethylene oxide)‐*block*‐poly(2‐(diethylamino)ethyl methacrylate)‐stat‐poly(methoxyethyl methacrylate) (Figure 3E).^[^ ^119^ ^]^ The results showed that the drug release rate of DOX‐loaded nanovesicles around the nucleus was observably accelerated under sonication, effectively inhibiting tumor growth in mice (tumor mass decreased by 95%) and minimizing systemic side effects. In comparison, unlike the physical rearrangement of such vesicles, the breakdown of intermolecular hydrogen bonds in vesicles assembled by ultrathin multiblock copolyamides also resulted in the release of hydrophobic drugs trapped in their cavities.^[^ ^120^ ^]^

Owing to their enhanced thermodynamic stability, polymer micelles have a more robust structure than their vesicle counterparts. As we described in the previous section, HIFU can convert hydrophobic THPMA units into hydrophilic MAA. Based on this, Zhao et al. prepared a polymer nanomicelle with poly(THPMA) as the core and poly(ethylene oxide) as the shell, which triggered the release of their molecular cargoes through phase transformation under US irradiation.^[^ ^121^ ^]^ Additionally, Xia et al. introduced mechanically unstable ester bonds into polymer micelles as US‐responsive mechanophores.^[^ ^122^ ^]^ Under HIFU irradiation, polymer micelles can be hydrolyzed and trigger the release of pyrene payloads, laying the foundation for the establishment of US‐responsive DDSs based on chemical bond transformation. Notably, the thermosensitive liposomes modified with copolymer poly(*N*‐isopropylmethacrylamide‐*co*‐*N*‐isopropylacrylamide) can release more than 60% of the encapsulated anticancer drugs upon US irradiation, which was attributed to the local hyperthermia (42 °C) caused by the rupture of acoustic cavitation bubbles.^[^ ^123^ ^]^

## 5 US‐CONTROLLED DRUG ACTIVATION

Although flourishing progress achieved, the conventional methods to control drug release are still facing several challenges, such as early drug leakage, high toxicity, and poor therapeutic efficacy. To this end, controlled drug activation strategies have been exploited recently to overcome these bottlenecks. This section will focus on the latest advances in the use of sonomechanical force to break covalent or non‐covalent bonds for controlled drug activation.

### 5.1 Covalent bond scission for drug activation

**FIGURE 4:**
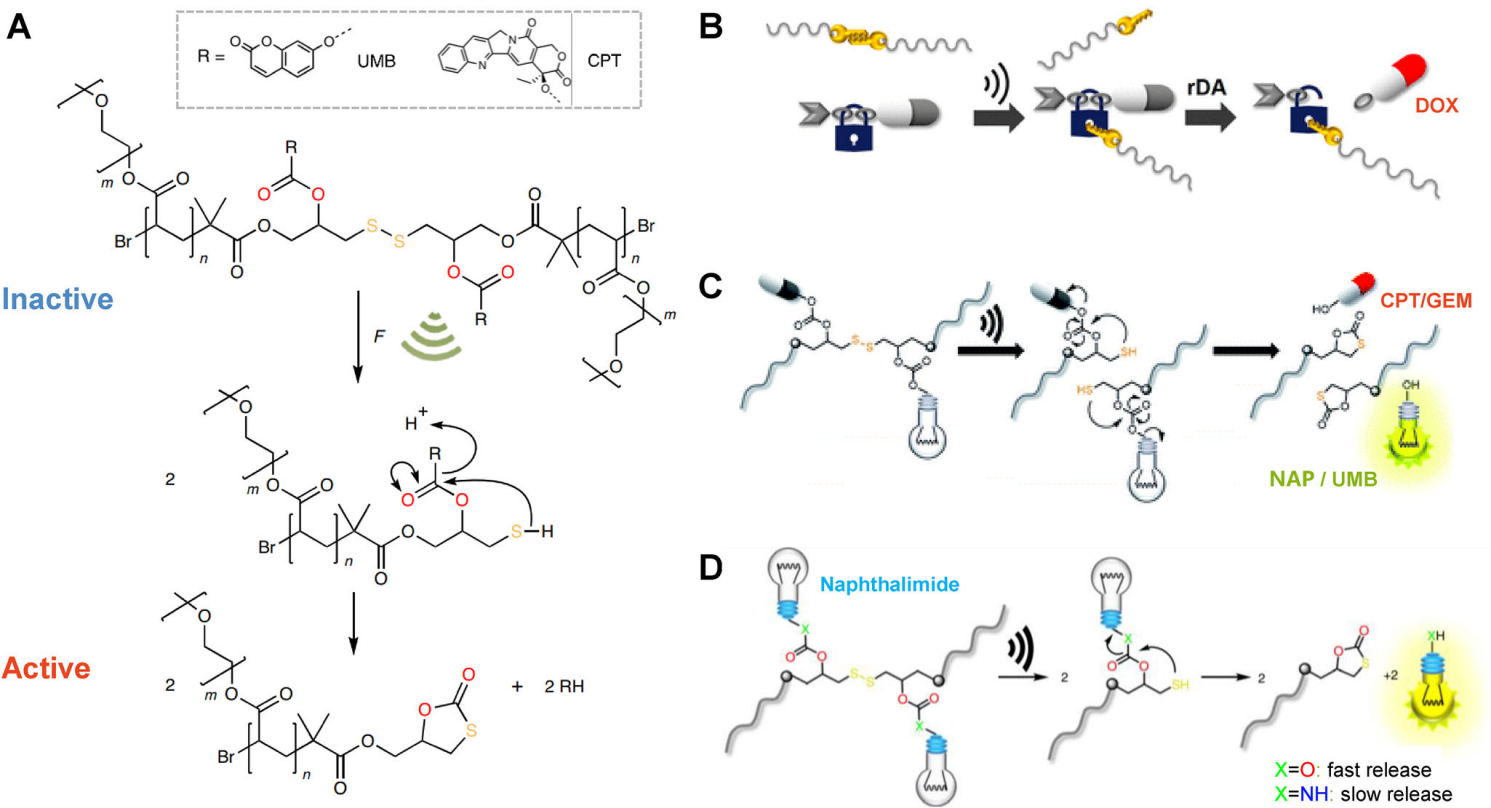
Representative examples of US‐controlled covalent bond scission for drug activation. (A) Ultrasonic cracking of the polymer centered on the S─S bond to activate the embedded CPT. Reproduced with permission.^[^ ^23^ ^]^ Copyright 2021, Springer Nature. (B) US‐induced fracture of polymer mechanochemical S─S bonds to activate drugs. Reproduced with permission.^[^ ^125^ ^]^ Copyright 2020, American Chemical Society. (C) Mechanochemical activation of disulfide‐based multifunctional polymers for theranostic drug release. Reproduced with permission.^[^ ^126^ ^]^ Copyright 2020, Royal Society of Chemistry. (D) Mechanochemical activation of the fluorophore from disulfide central polymers. Reproduced with permission.^[^ ^127^ ^]^ Copyright 2021, Chinese Chemical Society

In polymer mechanochemistry, the fracture of intramolecular covalent bonds will change the chemical properties of the molecule itself.^[^ ^124^ ^]^ To apply this concept to the drug activation systems, we recently realized sonomechanical force induced small‐molecule drug activation through the cleavage of covalent bonds within a polymer.^[^ ^23^ ^]^ In brief, two polymers centered with mechanically unstable S─S bonds were synthesized following the principles of mechanochemistry (Figure 4A). One was the polymer P_UMB_ labeled with fluorescent umbelliferone (UMB), and the other was the polymer P_CPT_ that carries the anticancer drug CPT. In theory, the functional center components of the polymer are inactivated because of the steric hindrance of the long‐chain polymer arms. Upon ultrasonication, the inactive components in P_UMB_ and P_CPT_ are activated by intramolecular 5‐exo‐trig cyclization caused by mechanical fracture of S─S bond. The results signified that the cell survival rate of P_CPT_ treated group was inversely proportional to the time of US irradiation, indicating the sonomechanical force treatment was positively correlated with the activation degree of CPT.

Likewise, an S─S bond was embedded in the center of the polymer chain that elicits the retro DA reaction by sonomechanical force, achieving the effective release and activation of conjugated furosemide and DOX (Figure 4B).^[^ ^125^ ^]^ To clarify the universality of the strategy, the activating candidates was further extended to a variety of amino‐ or hydroxy‐terminal drugs by combining the drug molecule with the β‐carbonate linker adjacent to the mechanically activated S─S bond, including CPT, *N*‐butyl‐4‐hydroxy‐1,8‐naphthalimide (NAP), gemcitabine (GEM) and UMB (Figure 4C).^[^ ^126^ ^]^ The indicative fluorescent reporting the functional molecules were successfully released and activated under US irradiation, confirming the feasibility and universality of regulating drug activity through chemical bond transformation. Most recently, we further explored the effect of β‐carbonate and ‐carbamate linkers on the mechanochemical responsiveness of disulfide‐centered polymers (Figure 4D).^[^ ^127^ ^]^ The results showed that hydroxy‐substituted NAP was effectively released from its β‐carbonate connectors within two days after US treatment. In contrast, the amino‐substituted NAP was released considerably slowly from its β‐carbamate linkers over several weeks, suggesting the leaving group properties of the respective amines were relatively poor. This study advances the exploration of force‐induce therapeutics with different release rates for further biomedical applications.

### 5.2 Non‐covalent bond scission for drug activation

**FIGURE 5:**
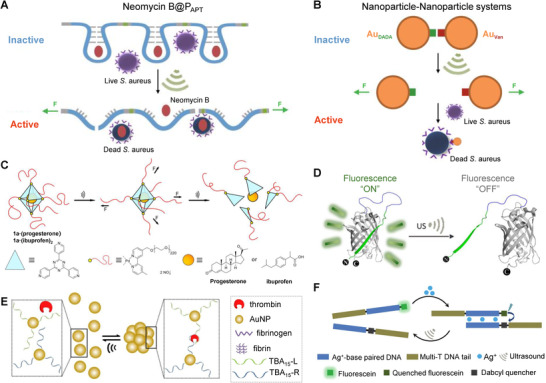
Representative examples of US‐controlled non‐covalent bond scission for drug activation. (A) The P_APT_ loaded with neomycin B releases their cargo through US‐induced stretching and bond scission. Reproduced with permission.^[^ ^23^ ^]^ Copyright 2021, Springer Nature. (B) Au NPs, DADA, and Van were assembled into Nanoparticle‐Nanoparticle systems to activate the antibacterial properties of Van. Reproduced with permission.^[^ ^23^ ^]^ Copyright 2021, Springer Nature. (C) Mechanochemical release of non‐covalently bound drugs from polymer‐grafted supramolecular cages. Reproduced with permission.^[^ ^129^ ^]^ Copyright 2021, John Wiley & Sons. (D) US‐induced unfolding of GFP modified by supercharged polypeptide. Reproduced with permission.^[^ ^131^ ^]^ Copyright 2020, John Wiley & Sons. (E) US induces reversible NP disaggregation leading to thrombin release for catalysis. Reproduced with permission.^[^ ^132^ ^]^ Copyright 2021, John Wiley & Sons. (F) US‐regulated dehybridization of metallo‐base paired DNA structures. Reproduced with permission.^[^ ^133^ ^]^ Copyright 2021, Royal Society of Chemistry

The scission of the covalent bond by sonomechanical forces is based on the destruction of the solid carrier‐cargo covalent interactions. In addition to covalent bonds, non‐covalent interactions are ubiquitous and alternative for constructing US‐sensitive drug activation systems.^[^ ^128^ ^]^ From this perspective, we proposed two strategies for activating drugs through the controlled breakage of non‐covalent bonds within macromolecules or nanocomponents.^[^ ^23^ ^]^ The first approach relies on the selective recognition of the RNA aptamer (APT) to its target molecule. As discussed above, the polymerization of aptamers (P_APT_) with high molecular weight provides a mechanical‐sensitive polymer carrier to bind the antibiotics and meantime inhibit their activities (Figure 5A). The infliction of sonomechanical force can impair the non‐covalent interactions, further breaking part of the covalent bond of the RNA backbone, thereby releasing and activating the drug molecule.

Inspired by the pharmacological action mechanism of vancomycin (Van), the second strategy relies on the supramolecular binding of Van with its targeting peptide sequence (DADA). First, we constructed a polymers‐NPs (PN) assembly by attaching DADA‐linked gold NPs (Au_DADA_) to Van‐terminated polymers (P_Van_) via hydrogen bond reciprocity. The results indicated that sonomechanical force could selectively break down multi‐hydrogen bonding in PN structures to release and activate drugs. Furthermore, to achieve a more effective drug activation response, Van‐coated NPs (Au_Van_) were synthesized, then assembled with Au_DADA_ into NPs‐NPs (NN) morphology (Figure 5B). Exhilaratingly, we found that, upon US treatment, the minimum inhibitory concentration (MIC) of NN was almost the same as that of free Van and was much lower than that of PN, indicating that the NPs‐based system has high ultrasonic sensitivity for drug activation. In another study, Schmidt et al. reported the absorption and release of non‐covalently encapsulated drugs in octahedral Pd cages with polymer chains anchored at each vertex (Figure 5C).^[^ ^129^ ^]^ The progesterone or ibuprofen was packaged and deactivated in a hydrophobic nano‐cavity of the supramolecular container. Since the star‐shaped structure is susceptible to the shear force, the encapsulated drug was wholly released when the coordinated nanocages were dissociated by US. The successful construction of non‐covalent US‐activated systems based on small molecule drugs also paves the way for the treatment of cancer and other diseases.

In addition to drug activation, there are several studies on the activity regulation of biomacromolecules through US‐induced non‐covalent bond dissociation, and their disorders and abnormalities are also closely associated with Alzheimer's disease, cardiovascular disease, and cancer.^[^ ^130^ ^]^ Recently, we demonstrated the first example of US‐controlled functional change of green fluorescent protein (GFP), quenching its fluorescence without changing the secondary structure of GFP (Figure 5D).^[^ ^131^ ^]^ After that, we designed two non‐covalent systems that can specifically activate the catalytic activity of thrombin by US.^[^ ^132^ ^]^ In this study, the experimental US (20 kHz) and clinical‐focused US (5 MHz) were used to selectively destroy the non‐covalent interaction between aptamer and thrombin, restoring thrombin activity and catalyzing fibrinogen to fibrin. More importantly, the ultrasonic response process of the NPs‐based system was completely reversible (Figure 5E). Thus, multiple cycles of US‐induced “inhibition‐activation” of the catalytic activity of thrombin can be realized. Besides, we recently reported the use of US to reversibly dehybridize the metallo‐base‐paired DNA structure, which provides a new strategy for remotely regulating the transformation and dynamic assembly of DNA structure (Figure 5F).^[^ ^133^ ^]^

## 6 CONCLUSION AND PROSPECTS

With the development of US‐responsive DDSs, US has gradually evolved into an on‐demand tool for remote drug release control in cancer therapy. Moreover, recent advances in US‐responsive DDSs have shown their great potential for the treatment of neurodegenerative diseases, diabetes, thrombosis, and COVID‐19.^[^ ^134^ ^]^ More importantly, sonomechanical forces have been shown to be capable of drug activation by selectively splitting mechanochemical bonds. An overview of representative stimuli‐responsive systems containing US‐sensitive components for controlling cargo release and activation was presented in Table 1. Without a doubt, these efforts are made possible by a thorough understanding of the various factors that influence the mechanochemical activity of carrier materials, which can help to accelerate the design and facilitate potential applications of US‐controlled DDSs.

**TABLE 1:** Summary of US‐sensitive components, action mechanism, and ultrasonication parameters of different US‐responsive systems discussed in the text

**US‐responsive system**	**US‐sensitive component**	**Action mechanism**	**US parameters**	**Ref**.
Azo‐functionalized PEG polymers	Azo bond	Mechanical effect	Freq.: 20 kHz Intens.: 8.7 W/cm^2^ Pulse (0.5 s on, 1 s off)	^[^ ^41^ ^]^
Chain‐centered coumarin dimer polymers	Coumarin dimer	Mechanical effect	Freq.: 20 kHz Intens.: 14.8 W/cm^2^ Pulse (1 s on, 1 s off)	^[^ ^65^ ^]^
Linear and three‐arm star polymers	Anthracene‐maleimide Diels‐Alder adduct	Mechanical effect	Freq.: 20 kHz Intens.: 13.8 W/cm^2^ Pulse (1 s on, 9 s off)	^[^ ^71^ ^]^
Poly(amide acid) vesicles	Non‐covalent bond	Mechanical effect	Freq.: 20 kHz Power: 37.5 W Duration: 3 min	^[^ ^81^, ^82^ ^]^
Diblock copolymer micelles	Anthracene‐maleimide Diels‐Alder adduct	Mechanical effect	Freq.: 20 kHz Pulse (1 s on, 2 s off)	^[^ ^83^ ^]^
Polymer grafted SiO_2_ NPs	Maleimide‐anthracene cycloadduct	Mechanical effect	Freq.: 20 kHz Pulse (0.5 s on, 1 s off)	^[^ ^86^, ^87^ ^]^
Microbubbles	Non‐covalent bond	Mechanical effect	Freq.: 400 kHz PRF: 1 Hz AP: 325 kPa Duration: 90 s	^[^ ^97^ ^]^
Microbubbles	Non‐covalent bond	Mechanical effect	Freq.: 1 MHz Intens.: 0.2 W/cm^2^ DC: 50%	^[^ ^102^ ^]^
Liposomes	Dipalmitoylphosphatidylcholine	Thermal effect	Freq.: 5 kHz DC: 30% Pulse (30 ms on, 70 ms off)	^[^ ^108^ ^]^
Liposomes	Non‐covalent bond	Mechanical effect	Freq.: 20 kHz Intens.: 3.3 W/cm^2^ Duration: 3 min	^[^ ^109^ ^]^
Liposomes	Non‐covalent bond	Mechanical effect	Freq.: 20 kHz Intens.: 5.9 W/cm^2^ Duration: 2 min	^[^ ^110^ ^]^
Polymer grafted MSNs	2‐tetrahydropyranic methacrylate	Thermal effect	Freq.: 1.3 MHz Power: 100 W Duration: 10 min	^[^ ^113^ ^]^
Hollow MSNs	Non‐covalent bond	Mechanical effect	Freq.: 1 MHz Intens.: 200 W/cm^2^ Duration: 100 min	^[^ ^114^ ^]^
Hollow MSNs	Non‐covalent bond	Mechanical effect	Freq.: 1 MHz Intens.: 1.5 W/cm^2^ DC: 50% Duration: 2 min Pulse (10 s on, 5 s off)	^[^ ^115^ ^]^
Ultrathin SiO_2_ coated nanoemulsions	Ultrathin SiO_2_ shell	Mechanical effect	Freq.: 3.5 MHz Power: 140 W Duration: 5 min	^[^ ^116^ ^]^
Diblock copolymer vesicles	Non‐covalent bond	Mechanical effect	Freq.: 40 kHz Power: 180 W	^[^ ^118^ ^]^
Diblock copolymer vesicles	Non‐covalent bond	Mechanical effect	Freq.: 1 MHz Intens.: 2.5 W/cm^2^ Duration: 3 min	^[^ ^119^ ^]^
Multiblock copolyamide vesicles	Hydrogen bond	Mechanical effect	Freq.: 40 kHz Power: 150 W	^[^ ^120^ ^]^
Diblock copolymer micelles	2‐tetrahydropyranic methacrylate	Thermal effect	Freq.: 1.1 MHz Power: 200 W	^[^ ^121^ ^]^
Diblock copolymer micelles	Ester bond	Mechanical effect	Freq.: 1.1 MHz Power: 150 W	^[^ ^122^ ^]^
Polymer modified liposomes	Poly(*N*‐isopropylmethacrylamide‐*co*‐*N*‐isopropylacrylamide)	Thermal effect	Freq.: 1 MHz Intens.: 0.5 W/cm^2^ DC: 100% Duration: 2 min	^[^ ^123^ ^]^
Disulfide‐centered polymers	Disulfide bond	Mechanical effect	Freq.: 20 kHz Duration: 240 min Pulse (2 s on, 1 s off)	^[^ ^23^ ^]^
Disulfide‐centered polymers	Disulfide bond	Mechanical effect	Freq.: 20 kHz Pulse (59 s on, 1 s off)	^[^ ^125^ ^]^
Disulfide‐centered polymers	Disulfide bond	Mechanical effect	Freq.: 20 kHz Intens.: 15.84 W/cm^2^ Duration: 180 min Pulse (2 s on, 1 s off)	^[^ ^126^ ^]^
Disulfide‐centered polymers	Disulfide bond	Mechanical effect	Freq.: 20 kHz Duration: 240 min Pulse (2 s on, 1 s off)	^[^ ^127^ ^]^
Neomycin B loaded polyaptamers	Hydrogen bond, electrostatic interaction, etc.	Mechanical effect	Freq.: 20 kHz Pulse (1 s on, 1 s off)	^[^ ^23^ ^]^
Au_Van_‐Au_DADA_ assemblies	Hydrogen bond	Mechanical effect	Freq.: 20 kHz Pulse (1 s on, 1 s off)	^[^ ^23^ ^]^
Polymer‐decorated supramolecular cages	Metal‐ligand bond	Mechanical effect	Freq.: 20 kHz Duration: 180 min Pulse (1 s on, 1 s off)	^[^ ^129^ ^]^
Supercharged polypeptide modified GFP	Hydrogen bond	Mechanical effect	Freq.: 20 kHz Intens.: 7 W/cm^2^ Pulse (2 s on, 1 s off)	^[^ ^131^ ^]^
Aptamer loaded with thrombin	Hydrogen bond, hydrophobic interaction, etc.	Mechanical effect	Freq.: 20 kHz Intens.: 10 W/cm^2^ Pulse (2 s on, 1 s off); Freq.: 5 MHz Duration: 6 min	^[^ ^132^ ^]^
Metallo‐base paired DNA structures	Metal‐base bond	Mechanical effect	Freq.: 20 kHz Duration: 20 min Pulse (1 s on, 1 s off)	^[^ ^133^ ^]^

Abbreviations: PRF: pulse repetition frequency, AP: acoustic pressure, DC: duty cycle.

Despite the unique advantages of using US to control drug release and drug activation, several challenges still need to be overcome before further clinic applications. One of the most critical issues is biosafety. As mentioned above, the extensive interplay of US with biological tissues may lead to undesirable safety risks. To this end, the FDA has formulated two criteria to quantify the tissue effects induced by US, namely thermal index (TI) and mechanical index (MI).^[^ ^135^ ^]^ The former represents the energy required to raise the tissue temperature by 1 °C, while the latter refers to a combination of ultrasonic parameters such as frequency and amplitude. On these grounds, current efforts should focus on the widespread use of US with high frequency, low amplitude, short irradiation time, and few duty cycles to achieve drug release and drug activity safely. From this point, the best option could be the clinical available medical US equipment. For example, clinically available HIFU has been attested recently to promote mechanochemistry conversion, underlining its clinical translational potential for US‐induced drug activation.^[^ ^136^ ^]^ Nevertheless, the optimal choice of ultrasonic parameters remains confusing due to the indisputable fact that the ideal purpose is to maximize the mechanical effects while minimizing adverse bioeffects. Therefore, developing safe and universal US guidelines for controlled drug release and drug activation remains a subject of future research.

Given that most of the currently reported US‐responsive DDSs are proof‐of‐concept studies, the following emphasis will be placed on bridging the gap from bench to bedside. Routinely, the construction of sonomechanical force‐activated DDSs counts on polymer chains, and its further applications are restricted by complex synthesis steps, low mechanochemical sensitivity, and poor drug delivery efficiency. As discussed above, several polymer‐NPs composite systems have been established to demonstrate their effective mechanochemical activation in vitro. One alternative direction could be the integration of mechanochemical polymer with nanomaterials to broaden their biomedical applications. Furthermore, highly aggregated NPs such as nanomicelles have higher mechanochemical activity than their stretched polymer counterparts. Combined with its preponderance with small size, we supposed that NPs could offer great potential for US‐induced drug delivery in the clinic.

Considering that nanomaterials have good biocompatibility, high drug loading, and accessible surface functionalization, novel US‐responsive nano‐DDSs with the simple preparation process and controllable release or activation of their cargo under clinically compatible US should be ulteriorly developed. Concomitantly, new force‐sensitive mechanophores should be explored and discovered to cross translational medicine boundaries to cater to diverse clinical demands.

In summary, compared with the traditional controlled drug release strategies, the research on US‐sensitive DDSs, especially the sonomechanial drug activation platform, is still in its infancy. Nonetheless, the superior properties and clinical translational potential make it of considerable value for widely biomedical applications. We believe that, with the continuous optimization of US‐sensitive DDSs, the side effects of chemotherapy drugs can be eliminated as much as possible. In the near future, we anticipate that more attempts based on US activation strategies will be successful and encouraging for cancer treatment, which will provide valuable references for improving the drug efficacy and reducing its associated side effects.

## CONFLICT OF INTEREST

There are no conflicts to declare.
